# Retinoid acid induced 16 deficiency aggravates colitis and colitis-associated tumorigenesis in mice

**DOI:** 10.1038/s41419-019-2186-9

**Published:** 2019-12-20

**Authors:** Yu-Lin Xu, Cui-Ling Ding, Chun-Lin Qian, Zhong-Tian Qi, Wen Wang

**Affiliations:** Department of Microbiology, Second Military Medical University, Shanghai, 200433 China

**Keywords:** Colorectal cancer, Chronic inflammation

## Abstract

Inflammatory bowel disease (IBD) and colitis-associated colorectal cancer (CAC) is a serious health issue, but etiopathological factors remain unclear. Although some studies reported the roles of Retinoid acid induced 16 (RAI16) in the tumorigenesis of hepatocellular carcinoma and PKA signaling, the roles of RAI16 in IBD and CRC are undressed. RAI16^−/−^ mice were generated and the roles of RAI16 were addressed in dextran sodium sulfate (DSS) or azoxymethane (AOM)-DSS induced IBD or CAC mouse models, respectively. At first, RAI16^−/−^ mice were viable, fertile with no apparent defects. Then, it was found that RAI16^−/−^ mice were more susceptibility to colitis induced by DSS than wild type (WT) littermates, which was evaluated by disease activity index and histological score. Furthermore, the expressions of tissues repair associated molecules Cox2, Ereg and MMP-10 were significantly decreased in RAI16^−/−^ colon under DSS treatment. Gut barrier related genes including antimicrobial peptides Reg3b and Reg3g and intestinal mucus genes Muc4, Muc6 and Muc20 were reduced in RAI16^−/−^ colon. These findings indicated that RAI16 may function to affect genes involved in intestinal barrier function and immunoprotective inflammation. Accordingly, RAI16^−/−^ mice displayed significantly increased tumor burden compared with WT mice assessed in CAC model induced by AOM/DSS. Much more Ki67 + nuclei were observed in RAI16^−/−^ tumors suggesting RAI16 to be critical in colonic cell proliferation during tumorigenesis. Conclusively, we demonstrate the roles of RAI16 in colonic inflammation and inflammation-associated tumorigenesis by using a novel RAI16^−/−^ mouse model for the first time.

## Introduction

Colitis-associated colorectal cancer (CAC) is one of the most commonly diagnosed and lethal cancer in developed countries^1,2^. More evidences suggest that excessive inflammatory condition in the gastrointestinal tract pose a high risk for CAC development^3^. Patients with inflammatory bowel disease (IBD) are at a higher risk of developing CAC^4^. IBD is a chronic inflammatory disease of the colon characterized by mucosal inflammation^5,6^. Dysfunctional mucus barrier of the epithelial cells lead persistently to the development of colonic inflammation, which can initiate genetic alterations of colonic epithelial cells, leading to neoplastic transformation, aberrant proliferation, angiogenesis and invasiveness, then to the development of tumorigenesis at last^7^. IBD pathogenesis and CAC tumorigensis are regarded as a multi-factorial and not completely understood^8^. Thus, it is still important to explore the molecular mechanisms of IBD and CAC progression.

Although the studies reported its role on cell proliferation and differentiation^9–11^, the functional study on RAI16 (also called FAM160B2) is limited. Previously, our group firstly reported that RAI16 enhanced tumorigenesis in hepatocellular carcinoma (HCC) due to the resistance to apoptosis and could serve as biomarker for HCC diagnosis^12^. Furthermore, we identified RAI16 as a novel A-kinase anchoring protein (AKAP), which regulated HSP70 associated anti-apoptosis signaling^13^. Homology analysis raveled that RAI16 is highly conserved in multispecies (human, mouse, rat, rabbit or zebrafish, et al.), suggesting that RAI16 might play important roles in cells.

In this study, we generated the RAI16 knockout (RAI16^−/−^) mouse model by CRISPR/Cas9 strategy, in order to evaluate the function of RAI16 by comparing RAI16^−/−^ mice with wild type mice in dextran sulfate sodium (DSS) induced colitis and azoxymethane (AOM)-DSS induced CAC mouse models. We demonstrated that RAI16^−/^^−^ mice were more susceptible to DSS induced colitis and CAC. The increase in tumorigenesis was related to cell proliferation in the colons of RAI16^−/−^ mice. Thus, these findings showed an important role for RAI16 in the pathogenesis of colitis and CAC.

## Materials and methods

### The generation of RAI16 knockout (RAI16^−/−^) C57BL/6 mice

Heterozygous RAI16^−/−^ C57BL/6 mice were generated using CRISPR/Cas9 strategy performed by CasGene Biotech Co., Ltd (BeiJing, China). The deletion of base pair in *RAI16* DNA or mRNA was confirmed by sequencing. The heterozygous pairs of RAI16^−/−^ mice were used to generate homozygous RAI16^−/−^ and littermate wild type mice for experimental studies. All animals were maintained by the Laboratory Animal Care Center of Second Military Medical University. All experiment procedures were approved by the Animal Research Committee of Second Military Medical University and all experiments were performed in accordance with relevant guidelines and regulations.

### DSS induced ulcerative colitis model

For generation of ulcerative colitis model, 18 RAI16^−/−^ mice and 18 wild type (WT) littermate mice (6–7 wk of age, bodyweight: 20–22 g) were given 3% DSS (wt/vol, MP Bio) for 6 days and then regular sterile water for 3 days. RAI16^−/−^ mice and wild type littermate mice in control groups were given regular sterile water for all 9 days. On the 9th day, all mice were sacrificed, the lengths of colons were measured and the colon was cut longitudinally with two distal 3-mm pieces preserved for further analysis.

### AOM-DSS induced CAC model

RAI16^−/−^ and WT mice were injected intraperitoneally with AOM (Sigma-Aldrich) at 7 mg/kg body weight. Five days later, these mice were given three cycles of 2% DSS for 5 days in sterile water, then 14 days regular sterile water. The body weight loss of these mice was monitored daily, and the mice with >20% body weight loss were considered dead and killed. After completion of the whole AOM-DSS regimen, these mice were sacrificed (at day 91), colons were removed and cut longitudinally. The number and size of tumors in colon of each mouse were blindly counted and measured.

### 16 S rDNA sequencing analysis of stool samples

16 randomly selected stool samples (8 samples from WT mice and 8 samples from RAI16^−/−^ mice) were stored until extraction at −20°. Approximately 200 mg of each stool sample was used for DNA extraction using Stool Mini Kit (Qiagen) according to the manufactures’. High-throughput was performed in Hiseq 2500 platform (Illumina) with Paired-End sequencing method (PE250) by the Beijing Genomics Institute (BGI, China). In brief, the 16 S rRNA gene with V4 regions was amplified with F515/R806 primers (GTGCCAGCMGCCGCGGTAA and GGACTACHVGGGTWTCTAAT). TruSeq® DNAPCR-Free Sample Preparation Kit was used to construct the amplicons libraries. The data retrieved was assembled and screened by Beijing Genomics Institute (BGI, China). The statistically gut microbial community composition differences and diversity indices between the samples of RAI16^−/−^ and WT mice were computed nonparametric unpaired *t*-test (*P* < 0.05) by using Microsoft Excel 2010 along with the aid of GraphPad Prism 7.

### Total RNA extraction and mRNA expression profiling

Total RNA of colon tissues was isolated as before. The RNA concentrations and the A260/A280 ratio were assessed with a multiplate reader (Synergy 2; BioTek, VT, USA). An A260/A280 ratio of 1.9 and a 28 S/18 S ratio of 1.8 were the minimum requirements for following the mRNA expression analysis. The mRMA expression levels of colon tissues from WT and RAI16^−/−^ mice (*n* = 5, respectively) were analyzed using the Illumina Mouse WG-6_v2 expression microarray (Illumina) platform by SHBIO, Co. (Shanghai, China). In brief, 500 ng of total RNA was amplified and biotin-labeled with the Illumina Total Prep-96 RNA Amplification Kit (Ambion, Austin, TX, USA). A total of 750 ng of labeled complementary RNA was hybridized to Illumina’s Mouse WG-6_v2 expression Bead Chips and then imaged using a Bead Array Reader according to manufacturer’s instructions. Data analysis was assessed by SHBIO, Co. (Shanghai, China) according to standard procedure. *P*-value < 0.05 was considered significantly different.

### The effects of *Akkermansia muciniphila* or ciprofloxacin

*Akkermansia muciniphila* (*A. muciniphila*) (ATCC BAA-835) was cultured in an anaerobic condition, according to ATCC culturing guidelines. Ciprofloxacin was purchased from Sigma-Aldrich. To examine the in vivo effects of *A. muciniphila* or ciprofloxacin on mice with colitis, RAI16^–/–^ and WT mice pretreated with *A. muciniphila* (6 × 10^8^ CFU/mouse) or ciprofloxacin (50 mg/kg/day) orally for 5 days, then administered by 3% DSS to for 6 days. DAI and histological score were used to evaluate the severity of disease of each mouse.

### Statistical analysis

GraphPad Prism7 was used for statistical tests. Two-tailed Student’s *t*-test was utilized to determine significant *p*-values for comparison of two groups. Individual *t*-tests were performed at each time point during DSS colitis. Log rank Mantel–Cox test was utilized for survival data. Differences were considered statistically significant when *P* ≤ 0.05. All data are presented as mean ± SD. *P* values are indicated by *P < 0.05, **P < 0.01 and ***P < 0.001.

## Results

### Generation and characterization of RAI16^−/−^ mice

**Fig. 1:**
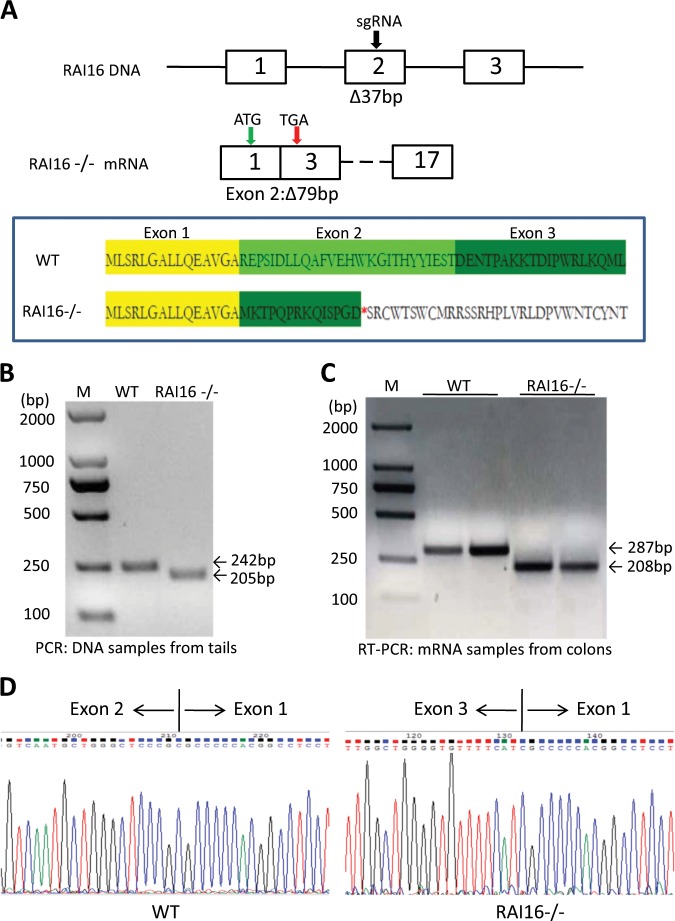
Targeted disruption of mouse *RAI16* gene. **a** Schematic representation of the gene targeting strategy for exon 2 of the *RAI16* gene. (A section of 37 bp was deleted in *RAI16* DNA, however, the whole exon 2 was skipped in *RAI16* mRNA). The red “*” represents the stop codon. **b** PCR analysis of *RAI16* DNA extracted from the tails of WT and RAI16^−/−^ mice. **c** RT-PCR analysis of *RAI16* mRNA extracted from the colon tissues of WT and RAI16^−/−^ mice. **d** RT-PCR products were confirmed by the sequencing.

To understand the exact biological functions of RAI16 in intestinal pathologies, we generated a RAI16^−/−^ mouse by using CRISPR-Cas9 strategy with the gRNA targeting the central section of exon 2 (Fig. 1a). All genotypes were determined by PCR using tail DNA. The mice with 37 bp deletion in exon 2 of *RAI16* were selected for further intercrossing (Fig. 1b). Unexpected, RT-PCR analysis indicated that the whole of *RAI16* exon 2 was deleted in tissues from colon (Fig. 1c, d). Thus, it was supposed that the exon 2 deletion would result in frame-shift, which would induce inactivation of RAI16 protein with a stop codon appearance in advance (Fig. 1a). However, no significant differences in protein level of RAI16 were observed upon deletion of *RAI16* exon 2 by Western blot with current commercial antibodies (Supplementary Fig. S1). Homozygous RAI16^−/−^ developed normally and no obvious phenotypic abnormalities were observed in RAI16^−/−^ mice compared with wild-type (WT) littermates up to 1 year of age.

### RAI16 knockout mice are more susceptible to DSS-induced colitis

**Fig. 2:**
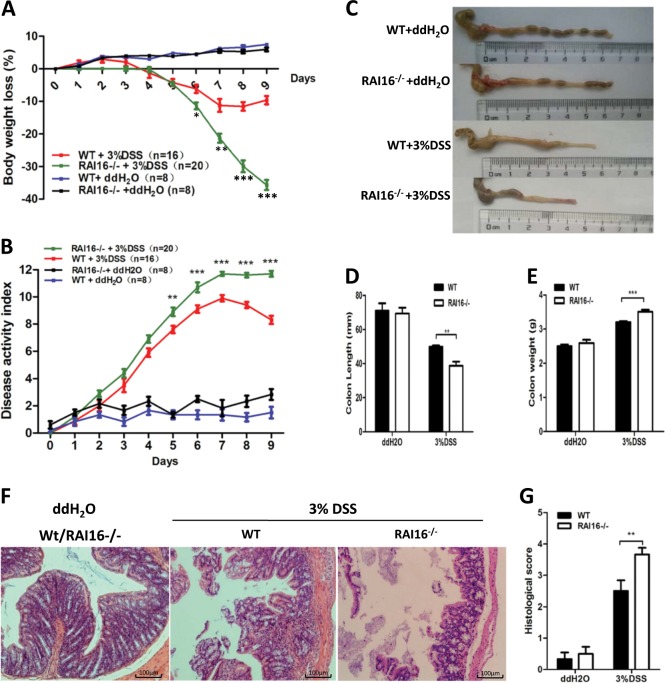
RAI16^−/−^ mice have more severe DSS induced colitis. **a** Body weight of WT and RAI16^−/−^ mice was determined daily. **b** Colitis severity was determined by disease activity index (including bleeding and diarrhea, etc.)on day 7. **c** The representatives of excised colons. **d** The lengths of excised colons. **e** The weights of excised colons. **f** Mucosal histology of the distal colon was examined on day 9 via H&E staining. **g** Histological scores were determined. Densitometric analysis for six independent experiments. Data shown are mean ± SD. ^*^p < 0.05, ^**^p < 0.01, ^***^p < 0.001.

To determine the role of RAI16 in mucosal immune responses, intestinal injury and inflammation using DSS colitis model was induced in WT and RAI16^−/−^ mice. To evaluate the severity of disease, body weight was measured and the diarrhea and fecal blood were monitored daily. Administration of 3% DSS for 6 days resulted in significant body weight loss in all mice, also diarrhea and rectal bleeding in some mice. In details, the body weight of WT mice started to decrease at day 4 and reached to ~12.8% decrease at day 7and then rebound later. Meanwhile, the body weight of RAI16^−/−^ mice also started to decrease at day 4, but accelerated decrease to ~20% decrease at day 7 and never rebound till the mice were killed at day 9 (Fig. 2a). No significance was found in WT or RAI16^−/−^ mice without DSS treatment. Consistently, DAI of RAI16^−/−^ mice was higher than that of the WT mice, starting from day 5 (Fig. 2b). Colon shortening was more severe in the RAI16^−/−^ mice (Fig. 2c). These results indicated that RAI16^−/−^ mice had greater weight loss (Fig. 2a), higher clinical scores (Fig. 2b) and shorter colon lengths (Fig. 2c-e) than WT mice, suggesting RAI16 deficiency exacerbates clinical and pathological symptoms in DSS-induced colitis mice model. Moreover, RAI16^−/−^ mice also displayed much more mucosal erosion, crypt destruction, goblet cells loss and inflammatory cell infiltration in the colon than WT mice (Fig. 2f, g). In addition, there is no significance between WT and RAI16^−/−^ mice for spontaneous colitis (data not shown). These data further indicated that RAI16^−/−^ mice were more susceptible to DSS-induced colitis.

### RAI16 knockout reduced the expression of repair-associated inflammatory cytokines during acute colitis

**Fig. 3:**
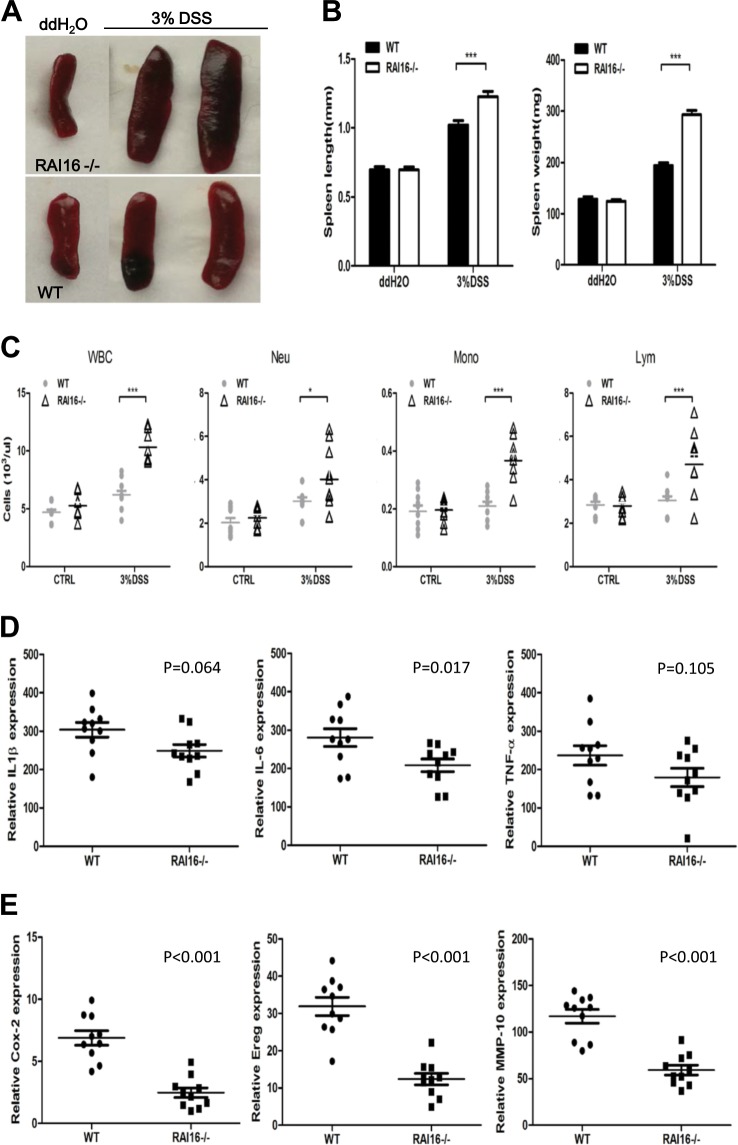
RAI16 knockout reduced the expression of repair-associated inflammatory cytokines in DSS induced colitis. **a** The spleens dissected from WT or RAI16^−/−^ mice on DSS for 7 days or water were imaged. **b** The lengths and weights of spleens of mice in A. **c** The cell numbers of neutrophil (Neu), monocyte (Mono), lymphocyte (Lym) and WBC from complete blood of mice in A. **d** The relative mRNA expressions of *IL-1β, IL-6* and *TNF-α* in colon tissues of mice treated with DSS were determined by qPCR. **e** The relative mRNA expressions of *Cox-2, Ereg* and *MMP-10* in colon tissues of mice treated with DSS were determined by qPCR. Data shown are mean ± SD. ***p < 0.001.

The systemic and local inflammatory response (including white blood cell elevation and spleen enlargement) in RAI16^−/−^ mice during colitis were examined. After 6-days DSS treatment, RAI16^−/−^ mice had enlarged spleens (Fig. 3a, b) and much more white blood cells than WT mice (Fig. 3c). The mRNA expression of several cytokines and chemokines in colonic tissues were measured using qRT-PCR. At the colitis induction stage (6-days of DSS treatment), no significance was observed between WT and RAI16^−/−^ mice. At the recovery stage (3-days of normal drinking water), the mRNA expressions of *IL-1β, IL-6* and *TNF-α* were significantly increased in DSS treated WT and RAI16^−/−^ mice (data not shown) as expected. However, the expression of *IL-6* mRNA in RAI16^−/−^ mice were lower than those in WT mice under DSS treatment, while the expression of *IL-1β* and *TNF-α* mRNA showed no significant between WT and RAI16^−/−^ mice under DSS treatment (Fig. 3d), which was inconsistent with the more severe colon injury in RAI16^−/−^ group.

There are other different mechanisms of RAI16 deficiency on DSS colitis. Then, mRNA expression of some repair-associated molecules in colon tissues were measured by quantitative RT-PCR. It was found that mRNA expressions of *Cox2, Ereg* and *MMP10* were significantly decreased in RAI16^−/−^ mice compared to that in WT mice under DSS treatment (Fig. 3e). No significance was found between WT and RAI16^−/−^ mice at baseline. Thus, the findings suggested RAI16 is most likely required not for tissue injury, but tissue repair in DSS-induced colitis.

### Deregulated expression of barrier associated genes in RAI16 knockout colon

**Fig. 4:**
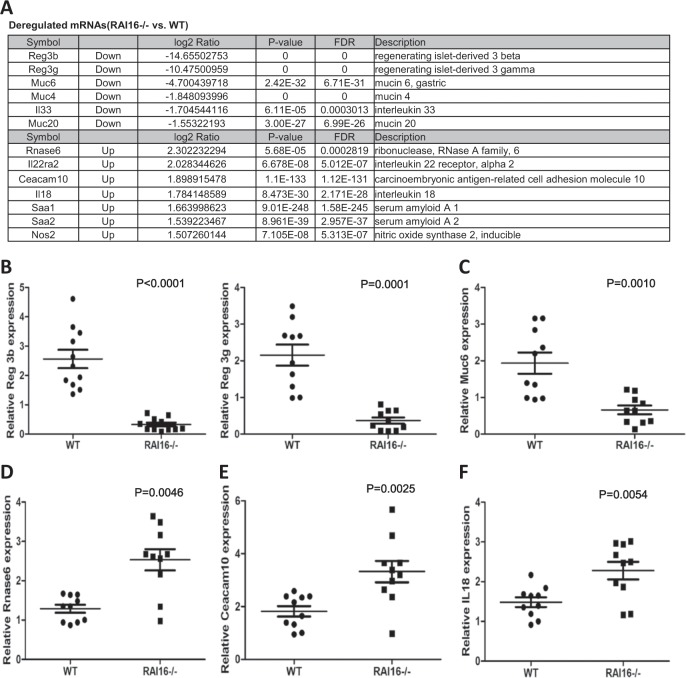
RAI16 knockout reduced the expression of genes important for intestinal barrier and inflammation cytokines. Mouse mRNA-chip was performed on a section of colon tissue of WT and RAI16^−/−^ mice. **a** The top deregulated mRNA for RAI16^−/−^ vs WT. **b-f** Select genes upregulated in RAI16^−/−^ colons, including *Reg3b* and *Reg3g* **b**, *Muc6* **c**, *Rnas6* **d**, *Ceacam10* **e**, and *IL-18* **f** were confirmed by qRT-PCR. Data shown are mean ± SD. *P* values were indicated.

In total, 304 upregulated genes and 409 downregulated genes (1.5 fold at least) were identified in colon tissues of RAI16^−/−^ mice vs. WT mice (Table S2). The top downregulated genes in RAI16^−/−^ colons were members of the C-type lectin antimicrobial peptide family *Reg3*: *Reg3b* and *Reg3g* (Fig. 4a, b). The mRNA of genes that produce intestinal mucus, including *Muc4, Muc6* (Fig. 4c) and *Muc20*, were also downregulated in RAI16^−/−^ colons. These results indicated that RAI16^−/−^ mice have reduced gut barrier function. However, other important barrier genes, including serum amyloid a 1 (*Saa1*) and *Saa2*, interferon response genes *RNase6* (Fig. 4d) and *Nos2*, as well as intestinal cell adhesion molecules *Ceacam10* (Fig. 4e) were expressed at higher levels in RAI16^−/−^ colon compared to WT. Also of note, the cytokine interleukin18 (*IL-18*) (Fig. 4f) and interleukin 22 receptor (*IL-22ra2*) were upregulated, but the cytokine interleukin33 (*IL-33*) was downregulated in the absence of RAI16 (representative data were showed in Fig. 4c–f). Thus, these findings demonstrated that RAI16 may function to affect those genes involved in barrier function and immune-inflammation of colons.

### Anti-bacilli treatment or supplement of *A. muciniphila* ameliorate DSS-induced colitis

**Fig. 5:**
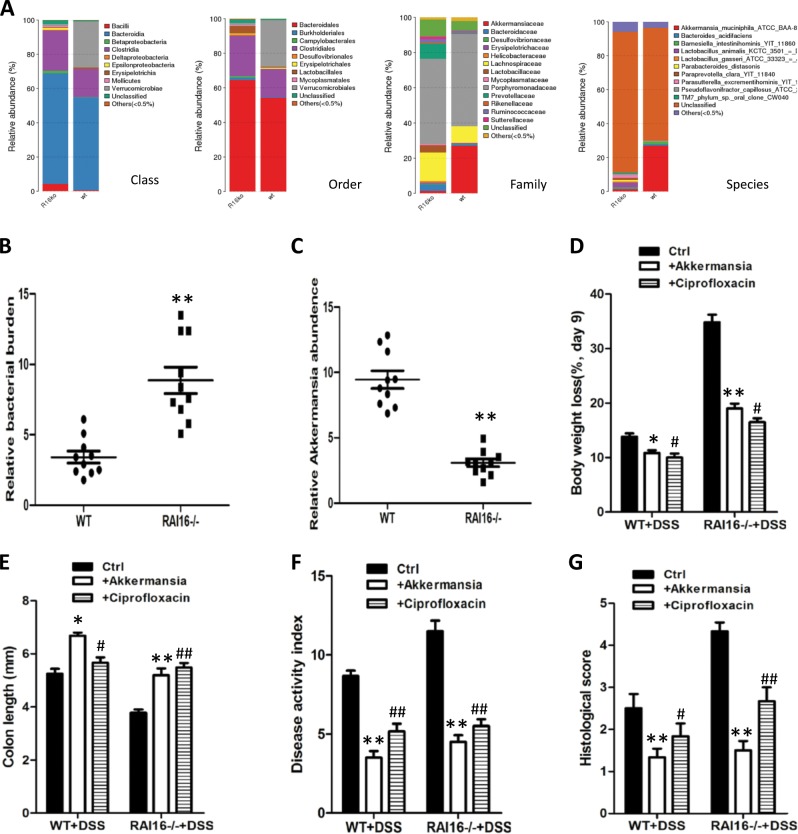
Anti-*bacilli* treatment or supplement of *A. muciniphila* ameliorate DSS-induced colitis in RAI16^−/−^ mice. **a** Gut microbiota levels of WT and RAI16^−/−^ mice. **b** Quantitative analysis of total gut bacterial burden in the feces of mice using qPCR. **c** Quantitative analysis of *bacilli* and *A. muciniphila* in the feces of mice using qPCR. WT and RAI16^−/−^ mice were treated ciprofloxacin oral or supplementated with *A. muciniphila* for 5 days and then given 3% DSS for 6 days and water for an additional 3 day. Severity of colitis were assessed by measuring body weights loss **d** and colon lengths **e**. Colitis severity was assessed using disease activity index **f** and histological scores **g**. Data represent one of three independent experiments. Data shown are mean ± SD. *p < 0.05; **p < 0.01; ^#^*p* < 0.05; ^##^*p* < 0.01.

*Firmicutes* and *Bacteroidetes* were the dominant phyla in all of the mice (Fig. 5a). Notably, *bacilli* were overexpresented in the fecal microflora of RAI16^−/−^ mice relative to their WT counterparts (Fig. 5a, b). Conversely, the RAI16^−/−^ mice had fewer bacteria of *A. muciniphila* compared with their respective WT littermates (Fig. 5a, c).

To determine whether an altered bacilli or *A. muciniphila* burden contributes to colitis severity in the RAI16^−/−^ colon, the mice were treated by ciprofloxacin or supplement of *A. muciniphila*. The DSS + *A.muciniphila* treated mice actually showed less weight loss while DSS treated mice showed more weight loss (Fig. 5d). DSS + *A.muciniphila* treated mice also had longer colons and lower DAI scores than DSS treated mice (Fig. 5e, f). Histological analysis showed that DSS + *A.muciniphila* treated colons had more epithelial stability and less inflammatory cell infiltration than DSS treated colons (Fig. 5g). Altogether, the administration of *A. muciniphila* could ameliorate the severity of DSS-induced colitis, playing protective functions in colons. On the other hand, the effects of anti-bacilli treatment on colitis severity in the RAI16^−/−^ colon were also determined. Supposedly, ciprofloxacin treatment could inhibit the proliferation of bacilli in RAI16^−/−^ colitis mice. As expected, ciprofloxacin treatment led to reduced weight loss (Fig. 5d), a longer colon length (Fig. 5e), and lower clinical and histological scores in RAI16^−/−^ mice (Fig. 5f, g). Taken together, these results further support the conclusion that an inability to control bacilli/*A. muciniphila* balance in colon leads to more severe colitis in RAI16 deficient mice.

### RAI16 knockout enhances development of CAC

**Fig. 6:**
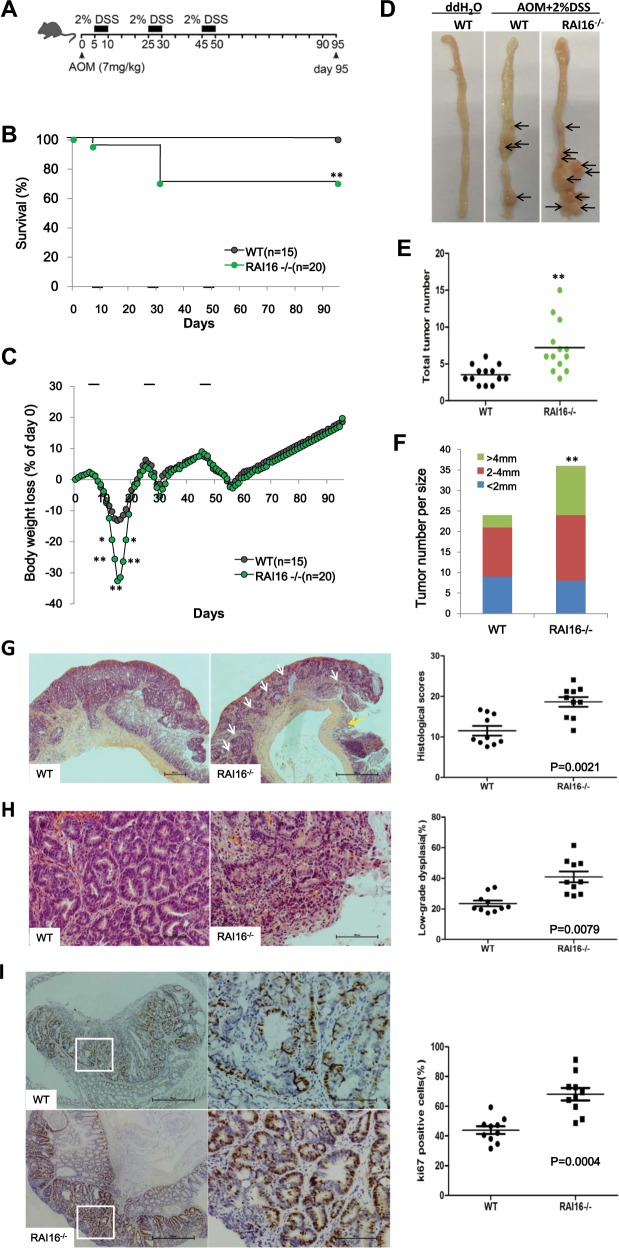
RAI16 deficiency promotes colitis-associated dysplasia progression. **a** Schematic representation of the AOM-DSS treatment. **b** Kaplan–Meier survival curve of WT (*n* = 15) and RAI16^−/−^ (*n* = 20) mice throughout the AOM-DSS regimen based on severe body weight loss as a consequence of excessive intestinal inflammation. P value was determined with Log-rank Mantel–Cox test. **c** Body weight loss of WT and RAI16^−/−^ mice treated as in **b**. **d** Photographs of gross representative appearance of the colons of WT and RAI16^−/−^ mice on day 95 post-treatment with AOM-DSS. **e** The average tumor numbers of WT and RAI16^−/−^ mice. **f** The average tumor sizes of in WT and RAI16^−/−^ mice. **g**, **h** Hematoxylin and eosin (H&E) staining of colon tissue section from WT and RAI16^−/−^ mice treated with AOM-DSS. Pathology scores evaluating colon tissue inflammation and ulceration of WT and RAI16^−/−^ mice were shown on the right panels **g**. Much more low-grade dysplasia was found in RAI16^−/−^ colon. The percentage of low-grade dysplasia was shown on the right panels **h**. **i** Colon sections were immunostained with anti-Ki67 antibody and Ki-67-positive cells were enumerated. Data represent mean ± SD of at least three independent experiments. *p < 0.05, **p < 0.01.

RAI16^−/−^ mice have been shown to be highly susceptible to DSS-induced colitis. It was prompted to explore the possible roles of RAI16 in the initiation and progression of colitis associated CAC. CAC was induced by azoxymethane (AOM)/DSS method as usual (single injection of AOM, then followed by three cycles of 2% DSS) in RAI16^−/−^ and WT mice respectively (Fig. 6a). Notably, RAI16^−/−^ mice were highly susceptible to colitis, as ~28% of RAI16^−/−^ mice died or exhibited dramatic body weight loss after only one cycle of DSS treatment (Fig. 6b, c). The mice that survived the first cycle of DSS-injury recovered and no further differences in body weight were observed between WT and RAI16^−/−^ mice throughout the remaining treatment period (Fig. 6c), probably due to enhanced proliferation of IECs with pro-tumorigenic capabilities in the absence of RAI16. Three months after AOM-DSS treatment, much more and larger tumors were developed in the middle and distal part of RAI16^−/−^ colons than WT colons (Fig. 6d–f). Thus, these findings indicated that RAI16 deficiency contributes to tumor development and progression in the colon, implying protective functions of RAI16 in CAC tumorigenesis.

Histopathological features of AOM-DSS induced CAC were assessed by H&E staining of colon tissue sections from WT and RAI16^−/−^ mice. RAI16 deficient colons had significantly more overall inflammation scores than WT colons (Fig. 6g). RAI16^−/−^ mice had a greater propensity to develop low-grade dysplasia in the inflamed epithelium (Fig. 6h). The staining of Ki-67 revealed much higher degrees of cell proliferation in colonic tumors in RAI16^−/−^ mice than that in WT mice (Fig. 6i). There is no evidence of distal metastasis in the lungs, spleen, liver or bone marrow found in WT or RAI16^−/−^ mice after the AOM-DSS treatment.

## Discussion

IL-6, IL-1β and TNF-α, which were reported to be involved in IBD commonly, have been widely used to evaluate the severity of IBD^14^. In the present study, *IL-6, IL-1β* and *TNF-α* mRNAs increased significantly in DSS treated WT and RAI16^−/−^ mice, however, there is no significance found between WT and RAI16^−/−^ mice under DSS treatment, even *IL-6* mRNA showed a bit of decrease in RAI16^−/−^ mice compared with WT mice. This finding was inconsistent with the increased colon injury in RAI16^−/−^ group. While tissues repair associated molecules were measured, it was found that the expression of *Cox2, Ereg* and *MMP-10* were significantly decreased in RAI16^−/−^ mice compared to that in WT mice under DSS treatment. Thus, RAI16 may play a role in tissue repair, but not tissue injury in DSS-induced colitis.

To explore the possible roles of RAI16, we compared the mRNA expressions of colon tissues between RAI16^−/−^ and WT mice. Firstly, *Reg3b* and *Reg3g* mRNAs were most significantly downregulated in RAI16^−/−^ colon tissues. Reg3, belongs to the C-type lectin antimicrobial peptide family, is expressed by IECs and functions to kill gram-positive bacteria^15,16^. Reg3b was reported to play essential roles in intestinal barrier function and protect mice from colitis^17,18^. Reg3g has also been reported to involve in intestinal barrier function^19^. The downregulation of Reg3b and Reg3g proteins in RAI16^−/−^ colon indicated that RAI16^−/−^ mice have reduced gut barrier function. Secondly, *IL-18* and *IL-22ra2* mRNAs were upregulted in RAI16^−/−^ colon tissues. Increased IL-18^20^ and IL-22ra2^21^ have been shown to play a crucial role in controlling tumorigenesis and epithelial cell proliferation in the colon. Moreover, the treatment of retinoic acid increased the production of Reg3β and Reg3γ in the colon, which are antimicrobial peptides responsive to IL-22^22^. In Stat3^ΔIEC^ mice, colonic expression of Stat3 target genes *Reg3β* and *Reg3γ* were significantly decreased, and the downregulation of *Reg3* mRNA expression may implicate impaired healing of the epithelium in colon^23^. Interesting, Rojo A Ratsimandresy RA, et al. have reported similar phenotype and possible mechanisms. In their study, Aim2^−/−^ mice also showed more susceptible to DSS induced colitis, which is mediated by the dysregulation STAT3 signaling and the IL-18/IL-22 dependent pro-proliferative and anti-microbial peptides of Reg3 family^19^. Thus, we hypothesis that RAI16 knockout could suppress the secretion of IL-18 and the expression of IL-22 binding protein (IL-22BP) in intestinal epithelial cells, consequently downregulate the secretion of STAT3-dependent Reg3γ and Reg3β, all together, which induces dysbiosis linked colitis. The third, the expression of *IL-33* was downregulated in RAI16^−/−^ colon tissues, suggesting the IL-33 related pathway might be involved in colitis process. Previously, Duan et al. has reported that the use of IL-33 ameliorated DSS induced colitis in mice by promoting regulatory T-cell responses^24^, while Pushparaj et al. demonstrated IL-33 exacerbated acute colitis via IL-4 in mice^25^; Zhu et al. simultaneously reported that IL-33 aggravated or alleviated DSS-induced acute colitis in mouse colon lamina propria by enhancing Th2 cell responses^26^ or by suppressing Th17 cell response as well as Th1 cell response^27^. Whether IL-33 or related pathway is involved in RAI16 deficiency associated colitis or CAC still needs further study.

In addition, the mRNA expressions of genes that produce intestinal mucus, including *Muc4*, *Muc6*, and *Muc20*, were downregulated in RAI16^−/−^ colons. The protective mucus barrier in gastrointestinal tract is remarkable, where the secretary and the membrane mucins form bi-layer together which provides protections by covering the epithelial cells^28^. Muc4^−/−^ mice are resistant to experimental colitis and colitis-associated colorectal cancer^29^; Higher Muc4 expression in early-stage CAC patients was related to poorer survival^30^; Muc5AC is associated with inflammation while Muc 6 is related to the presence of neoplasia^31^; Muc20 gene expression was found significantly decreased in patients with active UC^32^. Moreover, there are several other genes were dysregulated in RAI16^−/−^ colon, such as *Saa1, Saa2, RNase6, Nos2*, and *Ceacam10*. Saa1/2 has antibacterial effects and is involved in the protection from acute colitis^33^. RNase6 participates in the maintenance of urinary tract sterility as an antimicrobial peptide^34^; Marked increase of Nos2 expression was reported in colonic mucosa^35^; Ceacam20 was involved in colitis^36^ but Ceacam10 was firstly reported to be deregulated in DSS induced colitis model. These results suggest that RAI16 could be involved in the regulation of a range of important genes related on innate immune defense.

Recently, increasing evidences indicated that intestinal microbiota might play important roles in chronic inflammatory disease, including IBD^37^. Consistent with the previous studies^38^, DSS treatment resulted in a significant increase of *bacteroidetes* and *firmicutes* but a decrease of *verrucomicrobia*. Thus, RAI16 may be involved in the maintenance of colon health by preserving the microbial balance, stimulating the growth of beneficial bacteria but inhibiting the growth of pathogenic bacteria^39^.

Bacteria and their products play a crucial role in the pathogenesis of chronic intestinal inflammation in animal models and human IBD^40^. It was showed that the administration of ciprofloxacin improved colonic inflammation in RAI16^−/−^ mice. In addition, supplement of *A. muciniphila* could protect the progression of DSS-induced colitis. *A. muciniphila* is a gram negative anaerobe and belongs to *verrucomicrobia*. *A. muciniphila* can degrade highly glycosylated mucins proteins of intestinal epithelial mucus layer^41^. It was reported that the number of *A. muciniphila* was decreased in colon of IBD patients^42,43^. Also, it has been reported that extracellular vesicles derived from *A. muciniphila* could protect the progression of DSS-induced colitis^44^. The administration of *A. muciniphila* can re-establish the mucus layer in obese mice^45^. Moreover, Seregin et al. observed significantly increased levels of *A. muciniphila* in IL18^−/−^ mice and the administration of recombinant IL-18 (rIL-18) could reduce *A. muciniphila* colonization in Nlrp6^−/−^ mice^46^. These results indicate that IL-18 may modulate the relative abundance of *A. muciniphila*. Thus, RAI16 may regulate the abundance of *A. muciniphila* through IL-18 dependent pathway. Therefore, RAI16 not only plays a role in the regulation of interleukins related inflammation pathway, regulates the expression of serials of genes (*Reg3*, *Muc*, *Nos2*, et al.), but also has effect on the portion of intestinal microbiota. Aggravation of DSS-colitis by RAI16 deficiency must be a consequence influenced by multifactor and the mechanisms would be so complicated.

It was known that inflammation is correlated with tumorgenesis^47,48^. Increasing evidences have related the severity of colitis with the incidence of colorectal cancer^49,50^. In the present study, the AOM/DSS animal model has been used to demonstrate the importance of RAI16 in the development of colitis associated colon cancer. It was found that the number and the size of tumors in RAI16^−/−^ mice were higher compared to WT mice. In further, Ki67 strong staining indicated more robust cell proliferation in RAI16^−/−^ colon. Taken together, our finding suggests that the excessive immune response and cell repair/proliferation in RAI16^−/−^ mice may be the main cause of high colitis-associated cancer incidence.

RAI16 is conserved in several species by homology analysis, which indicated that RAI16 might play an important role in basic cell function. However, it has been difficult to study due to the absence of reliable antibody against RAI16 protein. Although the antibodies used in this study could recognize the overexpressed murine or human RAI16 protein well (data not shown), even the bands of “endogenous RAI16 protein” from mouse colon tissues could be eliminated by specific peptide competition, it was still hard to confirm the bands detected by antibodies are real RAI16 protein in Western blot (Fig. S1). These findings raised caution about the specificity of RAI16 antibodies. In fact, Western blot may not a reliable method for protein detection, because the quality of primary antibodies may be poor, as well as the different experimental conditions also affected the linearity and sensitivity of the assay. Weiqun et al. reported that all three commercially available antibodies against P2Y_6_ receptor recognized the same pattern of proteins in WT and knockout tissue by Western blot and no difference was also showed in staining patterns or intensity of knockout tissue sections by immunostaining^51^. Hafko et al. reported that identical binding patterns were detected in tissues of WT and angiotensin II (AT2) receptor knockout mice by three commercially available AT2 receptor antibodies^52^. Jensen et al. reported that none of the ten antibodies against alpha-1-adrenergic receptor subtypes (α1-ARs) from Abcam and Santa Cruz detected an appropriate band in WT but was absent in knockout tissues^53^. These reiterate that commercial antibodies need to be carefully validated before they can be correctly and effectively used. According to the importance of reliable antibodies for molecular research, we plan to prepare RAI16 specific antibodies by different peptides or recombinant protein in the future. We also plan to generate knockout mice with much larger section deletion (such as exon2 to exon 8, or exon 3 to exon 10 deletion).

In conclusion, for the first time we generated the RAI16 knockout mice and demonstrated that RAI16 has an important role in the colitis and CAC by regulating the expression of repair associated inflammatory cytokines, anti-bacterial peptides and microbes balance in colon. Next, the possible mechanism of RAI16 regulation would be addressed. There are some clues: (1) As a novel AKAP, RAI16 should be involved in much more PKA signaling related physiology or pathology process; (2) RAI16 is high expressed in thymus and CD4 + T cells, suggesting its role in immune regulation; (3) RAI16, also expressed in nucleus, may serve as a transcript factor regulating lots of genes expression.

## Supplementary information

Table S1

Table S2

Figure S1

Supplenmentary Materials and Methods

Supplementary Figure Legends

## References

[1] Baek SJ, Kim SH (2017). Colitis-associated colorectal cancer in patients with inflammatory bowel disease. Minerva Chir..

[2] Xue M, Shi L, Wang W, Chen S, Wang L (2018). An overview of molecular profiles in ulcerative colitis-related cancer. Inflamm. Bowel Dis..

[3] Rogler G (2014). Chronic ulcerative colitis and colorectal cancer. Cancer Lett..

[4] Fumery M (2017). Incidence, risk factors, and outcomes of colorectal cancer in patients with ulcerative colitis with low-grade dysplasia: a systematic review and meta-analysis. Clin. Gastroenterol. Hepatol..

[5] Waldner MJ, Neurath MF (2014). Mechanisms of immune signaling in colitis-associated cancer. Cell Mol. Gastroenterol. Hepatol..

[6] Sengupta N, Yee E, Feuerstein JD (2016). Colorectal cancer screening in inflammatory bowel disease. Dig. Dis. Sci..

[7] Ullman TA, Itzkowitz SH (2011). Intestinal inflammation and cancer. Gastroenterology.

[8] Pietrzyk L, Torres A, Maciejewski R, Torres K (2015). Obesity and obese-related chronic low-grade inflammation in promotion of colorectal cancer development. Asian Pac. J. Cancer Prev..

[9] Wen Y (2009). Loss-of-function mutations of an inhibitory upstream ORF in the human hairless transcript cause Marie Unna hereditary hypotrichosis. Nat. Genet..

[10] Xu W (2008). Prokaryotic expression and purification of retinoic acid induced 16 interacting with Tec kinase domain. World J. Gastroenterol..

[11] Luo XZ (2009). Construction of eukaryotic expression vector for RAI16 and its expression in HepG2 cells. ACTA Acad. Med Mil. Tertiae.

[12] Wang W (2012). Retinoic acid induced 16 enhances tumorigenesis and serves as a novel tumor marker for hepatocellular carcinoma. Carcinogenesis.

[13] Ding CL (2015). Anchoring of both PKA-RIIα and 14-3-3θ regulates retinoic acid induced 16 mediated phosphorylation of heat shock protein 70. Oncotarget.

[14] Sarra M, Pallone F, Macdonald TT, Monteleone G (2010). IL-23/IL-17 axis in IBD. Inflamm. Bowel Dis..

[15] Cash HL, Whitham CV, Behrendt CL, Hooper LV (2006). Symbiotic bacteria direct expression of an intestinal bactericidal lectin. Science.

[16] Kolls JK, McCray PB, Chan YR (2008). Cytokine-mediated regulation of antimicrobial proteins. Nat. Rev. Immunol..

[17] Brandl K, Plitas G, Schnabl B, DeMatteo RP, Pamer EG (2007). MyD88-mediated signals induce the bactericidal lectin Reg III gamma and protect mice against intestinal Listeria monocytogenes infection. J. Exp. Med..

[18] Vaishnava S (2011). The antibacterial lectin Reg III gamma promotes the spatial segregation of microbiota and host in the intestine. Science.

[19] Ratsimandresy RA, Indramohan M, Dorfleutner A, Stehlik C (2017). The AIM2 inflammasome is a central regulator of intestinal homeostasis through the IL-18/IL-22/STAT3 pathway. Cell Mol. Immunol..

[20] Zaki MH (2010). The NLRP3 inflammasome protects against loss of epithelial integrity and mortality during experimental colitis. Immunity.

[21] Huber S (2012). IL-22BP is regulated by the inflammasome and modulates tumorigenesis in the intestine. Nature.

[22] Mielke LA (2013). Retinoic acid expression associates with enhanced IL-22 production by γδ T cells and innate lymphoid cells and attenuation of intestinal inflammation. J. Exp. Med..

[23] Willson TA, Jurickova I, Collins M, Denson LA (2013). Deletion of intestinal epithelial cell STAT3 promotes T-lymphocyte STAT3 activation and chronic colitis following acute dextran sodium sulfate injury in mice. Inflamm. Bowel Dis..

[24] Duan L (2012). Interleukin-33 ameliorates experimental colitis through promoting Th2/Foxp3^+^ regulatory T-cell responses in mice. Mol. Med..

[25] Pushparaj PN (2013). Interleukin-33 exacerbates acute colitis via interleukin-4 in mice. Immunology.

[26] Zhu J (2015). IL-33 aggravates DSS-induced acute colitis in mouse colon lamina propria by enhancing Th2 cell responses. Mediators Inflamm..

[27] Zhu J (2015). IL-33 alleviates DSS-induced chronic colitis in C57BL/6 mice colon lamina propria by suppressing Th17 cell response as well as Th1 cell response. Int. Immunopharmacol..

[28] McGuckin MA, Linden SK, Sutton P, Florin TH (2011). Mucin dynamics and enteric pathogens. Nat. Rev. Microbiol.

[29] Das S (2016). Mice deficient in Muc4 are resistant to experimental colitis and colitis associated colorectal cancer. Oncogene.

[30] Shanmugam C (2010). Prognostic value of mucin 4 expression in colorectal adenocarcinomas. Cancer.

[31] Borralho P (2007). Aberrant gastric apomucin expression in ulcerative colitis and associated neoplasia. J. Crohns Colitis.

[32] Yamamoto-Furusho JK, Ascaño-Gutiérrez I, Furuzawa-Carballeda J, Fonseca-Camarillo G (2015). Differential expression of MUC12, MUC16, and MUC20 in patients with active and remission ulcerative colitis. Mediators Inflamm..

[33] Eckhardt ER (2010). Intestinal epithelial serum amyloid A modulates bacterial growth in vitro and pro-inflammatory responses in mouse experimental colitis. BMC Gastroenterol..

[34] Becknell B (2015). Ribonucleases 6 and 7 have antimicrobial function in the human and murine urinary tract. Kidney Int..

[35] Rafa H (2017). All-trans retinoic acid modulates TLR4/NF-κB signaling pathway targeting TNF-α and nitric oxide synthase 2 expression in colonic mucosa during ulcerative colitis and colitis associated cancer. Mediators Inflamm..

[36] Murata Y (2015). Intestinal cell adhesion molecules Protein tyrosine phosphatase SAP-1 protects against colitis through regulation of CEACAM20 in the intestinal epithelium. Proc. Natl Acad. Sci. USA.

[37] Ferreira CM (2014). The central role of the gut microbiota in chronic inflammatory diseases. J. Immunol. Res.

[38] Mar JS (2014). Amelioration of DSS-induced murine colitis by VSL#3 supplementation is primarily associated with changes in ileal microbiota composition. Gut Microbes.

[39] Duenas M (2015). A survey of modulation of gut microbiota by dietary polyphenols. Biomed. Res. Int..

[40] Podolsky DK (2002). Inflammatory bowel disease. N. Engl. J. Med..

[41] Derrien M, Vaughan EE, Plugge CM, de Vos WM (2004). Akkermansia muciniphila gen. nov., sp. nov., a human intestinal mucin-degrading bacterium. Int J. Syst. Evol. Microbiol.

[42] Png CW (2010). Mucolytic bacteria with increased prevalence in IBD mucosa augment in vitro utilization of mucin by other bacteria. Am. J. Gastroenterol..

[43] Rajilic-Stojanovic M, Shanahan F, Guarner F, de Vos WM (2013). Phylogenetic analysis of dysbiosis in ulcerative colitis during remission. Inflamm. Bowel Dis..

[44] Kang CS (2013). Extracellular vesicles derived from gut microbiota, especially Akkermansia muciniphila, protect the progression of dextran sulfate sodium-induced colitis. PLoS ONE.

[45] Everard A (2013). Cross-talk between Akkermansia muciniphila and intestinal epithelium controls diet-induced obesity. Proc. Natl Acad. Sci. USA.

[46] Seregin SS (2017). NLRP6 protects Il10–/– mice from Colitis by limiting colonization of Akkermansia muciniphila. Cell Rep..

[47] Mantovani A, Allavena P, Sica A, Balkwill F (2008). Cancer-related inflammation. Nature.

[48] Wang Z (2016). Oxidative stress and carbonyl lesions in ulcerative colitis and associated colorectal cancer. Oxid. Med Cell Longev..

[49] Itzkowitz SH, Yio X (2004). Inflammation and cancer IV. Colorectal cancer in inflammatory bowel disease: the role of inflammation. Am. J. Physiol. Gastrointest. Liver Physiol..

[50] Rutter M (2004). Severity of inflammation is a risk factor for colorectal neoplasia in ulcerative colitis. Gastroenterology.

[51] Yu WQ, Hill WG (2013). Lack of specificity shown by P2Y_6_ receptor antibodies. Naunyn Schmiedebergs Arch. Pharm..

[52] Hafko R (2013). Commercially available angiotensin II At(2) receptor antibodies are nonspecific. PLoS ONE.

[53] Jensen BC, Swigart PM, Simpson PC (2009). Ten commercial antibodies for alpha-1-adrenergic receptor subtypes are nonspecific. Naunyn Schmiedebergs Arch. Pharm..

