# Supplementary material for: Retinoid acid induced 16 deficiency aggravates colitis and colitis-associated tumorigenesis in mice
Source: Cell Death Dis. 2019 Dec 20;10(12):958. doi: 10.1038/s41419-019-2186-9 (PMC6925230; doi:10.1038/s41419-019-2186-9)
Supplement: Supplementary file 1 — Table S1 [file 41419_2019_2186_MOESM1_ESM.docx]

**Table S1. The primers in this study.**

| **Genes** | **Forward Primer(5’-3’)** | **Reverse Primer(5’-3’)** |
| --- | --- | --- |
| IL-6 | TCTTGGGACTGATGCTGGTG | TGCCATTGCACAACTCTTTTCT |
| IL-1β | TGTGAAATGCCACCTTTTGA | GGTCAAAGGTTTGGAAGCAG |

| TNF-a  Reg3b  Reg3g  Muc6  IL-18  Ceacam10  Rnase6 | cctctcatc agttctatggc  CTGCCTTAGACCGTGCTTTC  TTCCTGTCCTCCATGATCAAA  ctggggg cctatgccca tgc  GACCTGGAATCAGACAACTTTGG  CAGCCTCACTTTTAACTTACT  TGGCCCTGTTCA CCATAGGAGCC | GACAAGGTACAACCCATCG  ATAGGGCAACTTCACCTCAC  CATCCACCTCTGTTGGGTTCA  GCAGCCATCCACTGGCACAG  GCCTCGGGTATTCTGTTATGGA  GCACAGAAATCGGAGTAATT  GCGCATGGCTGTGTTGCATGG |
| --- | --- | --- |
| Cox-2 | CCCTTGGGTGTCAAAGGTAA | GCCCTCGCTTATGATCTGTC |
| Ereg | CTTCTACAGGCAGTTATCAGCAC | TCTCTCTCATGTCCACCAGGTAG |
| MMP-10 | AATTCAAGAAATGCAGAAGTTC | CACACTCTGTCTTGGCAAATC |
| GAPDH | CAATGAATAGGGCTACAGCA’ | AGGGAGATGCTCAGTGTTGG |
