# Supplementary material for: Retinoid acid induced 16 deficiency aggravates colitis and colitis-associated tumorigenesis in mice
Source: Cell Death Dis. 2019 Dec 20;10(12):958. doi: 10.1038/s41419-019-2186-9 (PMC6925230; doi:10.1038/s41419-019-2186-9)
Supplement: Supplementary file 2 — Table S2 [file 41419_2019_2186_MOESM2_ESM.docx]

**Table S2: The deregulated mRNAs of colon (RAI16-/- vs. WT)**

| **Symbol** | **log2 Ratio** | **P-value** | **FDR** | **Description** |
| --- | --- | --- | --- | --- |
| **Down** |  |  |  |  |
| Reg3b | -14.655 | 0 | 0 | regenerating islet-derived 3 beta |
| Tmem254b | -12.073 | 4.11E-177 | 5.58E-175 | transmembrane protein 254b |
| Mir5122 | -11.386 | 0.000136989 | 0.0006401 | microRNA 5122 |
| Reg3g | -10.475 | 0 | 0 | regenerating islet-derived 3 gamma |
| Fam177a | -10.156 | 1.94E-127 | 1.92E-125 | family with sequence similarity 177, member A |
| Xist | -9.462 | 0 | 0 | inactive X specific transcripts |
| Slc51a | -9.288 | 4.34E-28 | 1.05E-26 | solute carrier family 51, alpha subunit |
| Nts | -8.679 | 5.32E-15 | 7.05E-14 | neurotensin |
| Raet1e | -8.322 | 5.02E-12 | 5.46E-11 | retinoic acid early transcript 1E |
| Xlr3a | -7.738 | 3.02E-21 | 5.50E-20 | X-linked lymphocyte-regulated 3A |
| Gm14305 | -7.476 | 9.38E-09 | 7.68E-08 | predicted gene 14305 |
| 6230416C02Rik | -7.358 | 1.13E-06 | 7.21E-06 | zinc finger protein 973 |
| Tsix | -7.071 | 2.79E-32 | 7.68E-31 | X (inactive)-specific transcript, opposite strand |
| Pbp2 | -6.883 | 6.91E-05 | 0.0003382 | phosphatidylethanolamine binding protein 2 |
| Nags | -6.781 | 2.79E-32 | 7.69E-31 | N-acetylglutamate synthase |
| Ugt2b5 | -6.698 | 1.47E-58 | 7.51E-57 | UDP glucuronosyltransferase 2 family, polypeptide B5 |
| Gata4 | -6.687 | 7.77E-11 | 7.72E-10 | GATA binding protein 4 |
| Antxrl | -6.615 | 1.13E-06 | 7.20E-06 | anthrax toxin receptor-like |
| Duxbl2 | -6.476 | 1.13E-06 | 7.21E-06 | double homeobox B-like 2 |
| Duxbl3 | -6.476 | 1.13E-06 | 7.21E-06 | double homeobox B-like 3 |
| **Up** |  |  |  |  |
| Eif2s3y | 10.97656412 | 6.47E-105 | 5.21E-103 | eukaryotic translation initiation factor 2, subunit 3, structural gene Y-linked |
| Mptx1 | 9.945629973 | 0 | 0 | mucosal pentraxin 1 |
| Ddx3y | 9.831307244 | 1.69E-123 | 1.61E-121 | DEAD (Asp-Glu-Ala-Asp) box polypeptide 3, Y-linked |
| Hamp2 | 9.782998209 | 4.35E-11 | 4.43E-10 | hepcidin antimicrobial peptide 2 |
| Fxyd4 | 9.541096615 | 5.31E-12 | 5.77E-11 | FXYD domain-containing ion transport regulator 4 |
| Kdm5d | 9.424166289 | 4.28E-110 | 3.64E-108 | lysine (K)-specific demethylase 5D |
| Raet1d | 9.269126679 | 2.84E-23 | 5.67E-22 | retinoic acid early transcript delta |
| Casp14 | 9.214319121 | 6.09E-41 | 2.13E-39 | caspase 14 |
| 2310034C09Rik | 9.011227255 | 1.94E-14 | 2.49E-13 | RIKEN cDNA 2310034C09 gene |
| 2310079G19Rik | 8.948367232 | 6.47E-13 | 7.53E-12 | RIKEN cDNA 2310079G19 gene |
| 5033404E19Rik | 8.903881846 | 3.57E-10 | 3.34E-09 | NSA2 ribosome biogenesis homolog pseudogene |
| Pnliprp1 | 8.144658243 | 7.89E-14 | 9.70E-13 | pancreatic lipase related protein 1 |
| Speer5-ps1 | 7.851749041 | 8.01E-07 | 5.19E-06 | spermatogenesis associated glutamate (E)-rich protein 5, pseudogene 1 |
| H2-Q6 | 7.8008999 | 8.01E-07 | 5.18E-06 | histocompatibility 2, Q region locus 6 |
| 9030619P08Rik | 7.742013496 | 1.99E-187 | 2.86E-185 | lymphocyte antigen 6 complex pseudogene |
| Reg4 | 7.737094835 | 0 | 0 | regenerating islet-derived family, member 4 |
| Setdb2 | 7.426264755 | 2.37E-15 | 3.21E-14 | SET domain, bifurcated 2 |
| Psme2b | 7.409390936 | 1.32E-05 | 7.26E-05 | protease (prosome, macropain) activator subunit 2B |
| Uty | 7.388017285 | 2.28E-49 | 9.88E-48 | ubiquitously transcribed tetratricopeptide repeat gene, Y chromosome |
| Mettl7a3 | 7.375039431 | 1.94E-14 | 2.49E-13 | methyltransferase like 7A3 |
