# Supplementary figures and images for: Retinoid acid induced 16 deficiency aggravates colitis and colitis-associated tumorigenesis in mice
Source: Cell Death Dis. 2019 Dec 20;10(12):958. doi: 10.1038/s41419-019-2186-9 (PMC6925230; doi:10.1038/s41419-019-2186-9)

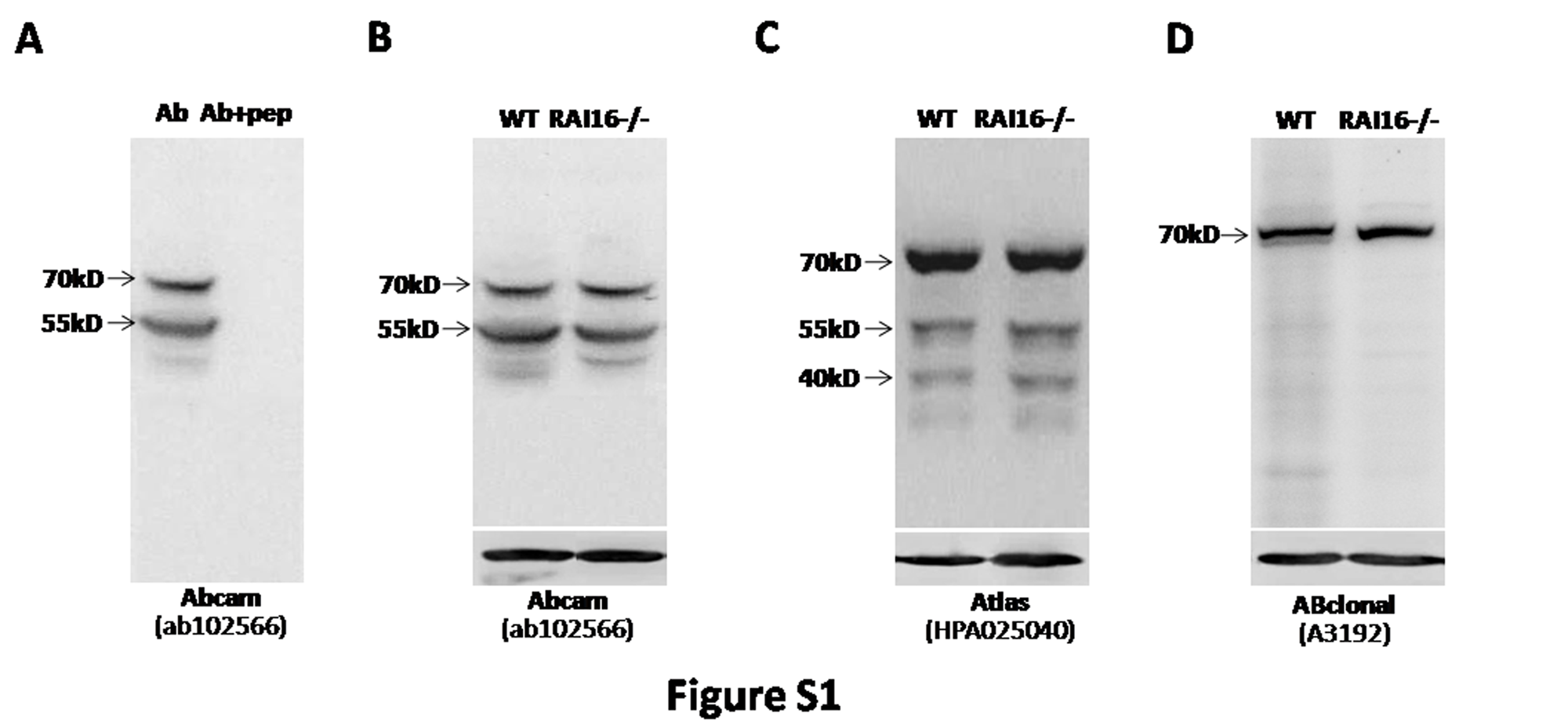

Supplement: Supplementary file 3 — Figure S1 [file 41419_2019_2186_MOESM3_ESM.tif]
