# Supplementary material for: Retinoid acid induced 16 deficiency aggravates colitis and colitis-associated tumorigenesis in mice
Source: Cell Death Dis. 2019 Dec 20;10(12):958. doi: 10.1038/s41419-019-2186-9 (PMC6925230; doi:10.1038/s41419-019-2186-9)
Supplement: Supplementary file 4 — Supplenmentary Materials and Methods [file 41419_2019_2186_MOESM4_ESM.docx]

**Supplymentary materials**

**Materials and methods**

**Disease activity index (DAI)**

The mice were monitored daily or weekly during the course of colitis or CRC development. The disease activity of colitis was scores as described previously (1), and these scores of body weight loss, diarrhea and bleeding were summarized as DAI.

**Histopathology**

Colon tissues were fixed in 10% formalin overnight, embedded in paraffin and cut in 4μm sections. After staining with Hematoxylin & eosin (H&E), sections were scored for the inflammation and ulcerations blindly by at least 3 gastrointestinal pathologists according to the criteria as described previously (2). Low (LGD) or high (HGD) grade of colonic mucosal dysplasia were defined as previously described criteria (3).

**Immunohistochemistry**

After dewaxing and hydration, paraffin sections were boiled in citrate for antigen retrieval. Endogenous peroxidase activity was blocked by 3% hydrogen peroxide for 15 min at room temperature. The slides were blocked by incubating in 5% bovine serum albumin (BSA) at 37 °C for 30 min. Sections were then incubated with primary antibodies against Ki-67 (1:100 dilution, CST) overnight at 4°C, followed by incubation with HRP-conjugated goat anti-rabbit antibody (1:1000 dilution, KPL) for one hour at room temperature. Then, the sections were incubated with 3, 3’-diaminobenzidine tetrahydrochloride (DAB) for signal development. Finally, the slides were analysed by at least three pathologists blindly.

**Quantitative real-time RT-PCR**

Total RNA was isolated from colon tissues using TRI Reagent combined with RNeasy Tissue kit (Qiagen) according to the manufacturer’s recommendations. The following reverse transcriptase reaction contained 100 ng RNA, 3U/μL reverse transcriptase 0.25U/μL, 50 nmol/L primers and other regular reagents. Then, the qRT-PCR reactions including 2μL cDNA product, 10uL SYBR Green Supermix and 1μL gene-specific primers were performed as following procedure: 95°C for 5 min, followed by 40 cycles at 95°C for 30 sec and 58°C for 45 sec. The data were analyzed using the 2^–ΔΔCt^ method and presented as the fold change in RAI16-/- group relative to WT group after normalized to the expression of GAPDH. Primer sequences used in this study are summarized in Table S1.

**References**

1. Danese, S., Malesci, A. & Vetrano, S. Colitis-associated cancer: the dark side of inflammatory bowel disease. *Gut* **60**, 1609–1610(2011).

2. Meira, L.B. et al. DNA damage induced by chronic inflammation contributes to colon carcinogenesis in mice. *The Journal of clinical investigation* **118**, 2516–2525(2008).

3. Rubio, C.A. & Delinassios, J.G. Invasive carcinomas may arise in colorectal adenomas with high-grade dysplasia and with carcinoma in situ. *Int J Clin Exp Med* **3**,41-47(2010).
