# Supplementary material for: Retinoid acid induced 16 deficiency aggravates colitis and colitis-associated tumorigenesis in mice
Source: Cell Death Dis. 2019 Dec 20;10(12):958. doi: 10.1038/s41419-019-2186-9 (PMC6925230; doi:10.1038/s41419-019-2186-9)
Supplement: Supplementary file 5 — Supplementary Figure Legends [file 41419_2019_2186_MOESM5_ESM.docx]

**Supplementary Figure legends**

**Figure S1. The detection of RAI16 protein by Western blot.**

**A.** Proteins extracted from colon tissues of WT mice were detected for RAI16 by Western blot with a commercial antibody from Abcam (ab102566). The peptide competitive inhibition assay was performed by using a peptide of RAI16 N terminal amino acids (35-84: HYYIESTDESTPAKKTDIPWRLKQMLDILVYEEQQQAAAGEAGPCLEYLL) as regular protocol. **B-D.** Proteins extracted from colon tissues of RAI16-/- and WT mice was detected for RAI16 by Western blot with indicated commercial antibodies from Abcam (ab102566), Atlas (HPA025040) and Abclonal (A3192), respectively. Pep: peptide.
